# p62-Nrf2 Regulatory Loop Mediates the Anti-Pulmonary Fibrosis Effect of Bergenin

**DOI:** 10.3390/antiox11020307

**Published:** 2022-02-03

**Authors:** Qian Zeng, Tingting Zhou, Feiyan Zhao, Dayan Xiong, Bin He, Qingzhong Hua, Miao Lin, Lang Deng, Xiaoxue Sang, Weixi Xie, Jia Chen, Zun Wang, Lu Ren, Ziqiang Luo, Xiaoting Huang, Wei Liu, Siyuan Tang

**Affiliations:** 1 Department of Community Nursing, Xiangya Nursing School, Central South University, Changsha 410013, China; zq18373153202@163.com (Q.Z.); Z18434160983@163.com (T.Z.); xiongdayan@126.com (D.X.); huaqz920921@163.com (Q.H.); lin1999@csu.edu.cn (M.L.); dengL036@163.com (L.D.); sangxiaoxue21@163.com (X.S.); 18508450233@163.com (W.X.); Wang_zun@csu.edu.cn (Z.W.); renlu108811@csu.edu.cn (L.R.); 2 College of Veterinary Medicine, Hunan Agricultural University, Changsha 410125, China; zhaofeiyan2018@hunan.edu.cn; 3 School of Nursing, Hunan University of Medicine, Huaihua 410082, China; hebin2008082022@163.com; 4 Hunan Provincial Key Laboratory of Nursing, Xiangya Nursing School, Central South University, Changsha 410013, China; hlxycjia@csu.edu.cn; 5 Department of Physiology, Xiangya School of Medicine, Central South University, Changsha 410013, China; luoziqiang@csu.edu.cn; 6 Chronic Disease Basic Research and Health Management Laboratory, Xiangya School of Medicine, Central South University, Changsha 410013, China

**Keywords:** bergenin, oxidative stress, nuclear factor erythroid 2-related factor 2, p62/SQSTM1, idiopathic pulmonary fibrosis, myofibroblast

## Abstract

Idiopathic pulmonary fibrosis (IPF) can severely disrupt lung function, leading to fatal consequences, and there is currently a lack of specific therapeutic drugs. Bergenin is an isocoumarin compound with lots of biological functions including antioxidant activity. This study evaluated the potential beneficial effects of bergenin on pulmonary fibrosis and investigated the possible mechanisms. We found that bergenin alleviated bleomycin-induced pulmonary fibrosis by relieving oxidative stress, reducing the deposition of the extracellular matrix (ECM) and inhibiting the formation of myofibroblasts. Furthermore, we showed that bergenin could induce phosphorylation and expression of p62 and activation of Nrf2, Nrf2 was required for bergenin-induced p62 upregulation, and p62 knockdown reduced bergenin-induced Nrf2 activity. More importantly, knockdown of Nrf2 or p62 could abrogate the antioxidant activity of bergenin and the inhibition effect of bergenin on TGF-β-induced ECM deposition and myofibroblast differentiation. Thereby, a regulatory loop is formed between p62 and Nrf2, which is an important target for bergenin aimed at treating pulmonary fibrosis.

## 1. Introduction

Pulmonary fibrosis describes a condition in which the normal lung anatomy is replaced by a process of active remodeling, extracellular matrix deposition, and myofibroblastic foci formation. The disease can result from environmental exposure, severe infection, chemotherapy, radiation, or unknown etiologies, as in idiopathic pulmonary fibrosis (IPF). IPF is a progressive, and ultimately fatal, respiratory disorder that represents the most common, idiopathic form of pulmonary fibrosis [1]. Although the disease course of IPF is variable and somewhat unpredictable, the median survival time from diagnosis is about 3–5 years [2]. For the treatment of IPF, the only drugs recommended by clinical guidelines and approved by the United States Food and Drug Administration (FDA) are pirfenidone and nintedanib, but the therapeutic effects of pirfenidone and nintedanib are limited and accompanied by many adverse reactions and side effects [3]. Thus, there is an urgent need to identify new effective drugs for the treatment of pulmonary fibrosis.

Oxidative stress refers to the generation of excessive reactive oxygen species (ROS) and/or the depletion of antioxidant defenses caused by abnormal molecules, cells, or tissues. A variety of biological molecules in the body’s cells may be destroyed under oxidative stress [4,5]. The lungs are exposed to the highest levels of oxygen in the human body over a prolonged period; therefore, they are more susceptible than other organs to oxidative stress [6]. Accumulating evidence suggests that oxidative stress plays an essential role in the pathogenesis of pulmonary fibrotic disorders [7]. As an important pathogenetic mechanism, oxidative stress can promote fibrosis by increasing the transformation of fibroblasts to myofibroblast [8], inducing apoptosis of alveolar epithelial cells, and upregulating the expression of profibrotic cytokines (including TGFs) [9,10]. Moreover, studies have shown that inhibiting oxidative stress can effectively reduce pulmonary fibrosis [11,12].

Transforming growth factor-β (TGF-β) is the most important cytokine promoting the occurrence and development of pulmonary fibrosis, and it can promote the transformation of fibroblast to myofibroblast and the increase of extracellular matrix protein [13,14]. Fibroblasts, macrophages, and epithelial cells secrete significant amounts of TGF-β after lung tissue is exposed to bleomycin, paraquat, and silicon dioxide, and the activated TGF-β regulates the expression of profibrotic genes [15,16]. Although the specific mechanisms have not been fully ascertained, increasing evidence shows that oxidative stress plays a central role in TGF-β-related fibrosis. ROS can stimulate the expression and secretion of TGF-β in a variety of cells in addition to activating latent TGF-β [17,18,19,20,21,22]. Furthermore, ROS can promote the phosphorylation of Smad [23,24] and then activate Smad2/3 to mediate the differentiation of myofibroblasts induced by TGF-β1 [25]. In turn, TGF-β can directly stimulate the production of ROS through Nox4 and mitochondria [26,27]. TGF-β can also increase the ROS level by inhibiting the expression of several antioxidant enzymes [28,29,30]. The relationship between TGF-β and ROS forms a vicious circle aggravating fibrosis.

To prevent damage caused by oxidative stress, many substances in the body will play a role in antioxidant defense, and among these, nuclear factor erythroid 2-related factor 2 (Nrf2) is a main regulatory factor [31]. In mouse models, Nrf2-knockout mice are more sensitive to pulmonary fibrosis induced by bleomycin and paraquat than wild-type mice [32,33]. In vitro, cultures of primary lung fibroblasts obtained from controls or IPF patients showed that decreased Nrf2 expression is associated with an increased expression of myofibroblast phenotypes (type I collagen and α-SMA), and the activation of Nrf2 increased antioxidant defenses and decreased the expression of myofibroblast phenotypes [34]. These findings suggest that Nrf2 may be a therapeutic target for IPF and other fibrotic diseases caused by oxidative stress. 

Bergenin is an isocoumarin compound, which was first extracted from plants of the genus *Bergenia* [35]. Bergenin exerts a strong antioxidant effect [36,37]. Considering the role of oxidative stress in the development of pulmonary fibrosis, our study will assess the potential therapeutic effect of bergenin on pulmonary fibrosis and investigate its possible mechanisms. For the first time, we proved that bergenin exerts a direct anti-pulmonary fibrosis effect, which is mediated by activating the positive feedback loop of p62–Nrf2.

## 2. Materials and Methods

### 2.1. Experimental Animals

Male C57BL/6 mice and SD rats were ordered from the Animal Center of Central South University. On day 0, an intratracheal injection of 50 μL of 3.3 U/kg of bleomycin (Nippon Kayaku, Tokyo, Japan) or an equivalent amount of normal saline was administered, following pentobarbital sodium anesthesia. Normal saline, containing 40% PEG300 (Macklin, Shanghai, China), 5% dimethyl sulfoxide (DMSO) (Macklin, Shanghai, China), and 5% Tween80 (Macklin, Shanghai, China) (*v*/*v*/*v*), was used as the vehicle to dilute bergenin (Selleck Chemicals, Houston, Texas, USA). The vehicle and bergenin treatments were injected intraperitoneally.

The experimental plan was divided into the treatment and inhibitor plans. The treatment plan included 110 mice, among which 10 were randomly selected as controls, and the rest were used for bleomycin modeling. The intratracheal injection of normal saline on day 0 was followed by an intraperitoneal injection of the vehicle on days 15–28 in the control group. The intratracheal injection of bleomycin was administered on day 0, and the successfully modeled mice were randomly divided into four equal groups on day 14: (1) the pulmonary fibrosis model group with an intraperitoneal injection of the vehicle on days 15–28; (2) the high-dose group with an intraperitoneal injection of 25 mg/kg of bergenin on days 15–28; (3) the moderate-dose group with an intraperitoneal injection of 5 mg/kg of bergenin on days 15–28; and (4) the low-dose group with an intraperitoneal injection of 1 mg/kg of bergenin on days 15–28. On day 29, all mice were euthanized, and the lung tissue was removed. The inhibitor plan included 100 mice, among which 10 were randomly selected as controls, and the rest were used for bleomycin modeling. The intratracheal injection of normal saline on day 0 was followed by an intraperitoneal injection of the vehicle on days 15–28 in the control group. The intratracheal injection of bleomycin was administered on day 0, and the successfully modeled mice were randomly divided into three equal groups on day 14: (1) the pulmonary fibrosis model group with an intraperitoneal injection of the vehicle on days 15–28; (2) the high-dose group with an intraperitoneal injection of 25 mg/kg of bergenin on days 15–28; and (3) the inhibitor group with an intraperitoneal injection of 30 mg/kg of ML385, 3 h before the intraperitoneal injection of 25 mg/kg of bergenin, on days 15–28. All mice were raised in a specific pathogen-free level environment and subjected to normal day and night rhythms, and sufficient food and water were provided for their daily needs. Various measures were taken to reduce the pain of mice during the entire experiment. This study was conducted in accordance with the welfare and ethical principles of laboratory animals. The experimental protocol was approved by the Laboratory Animal Welfare and Ethical Committee of Central South University (IACUC number: 2021sydw0164). 

### 2.2. Cell Culture

All cells were cultured in a humid environment containing 5% carbon dioxide at 37 °C. Mouse embryonic fibroblasts (NIH3T3 cells) were obtained from the State Key Laboratory of Genetics (Changsha, China) and cultured in Dulbecco’s modified Eagle medium (DMEM)–high glucose (Procell, Wuhan, China) containing 10% newborn bovine serum (NBS, Sigma, St. Louis, MO, USA) and 1% penicillin–streptomycin solution (Gibco, Waltham, MA, USA). Human lung fibroblasts (HFL-1 cells) were obtained (Procell, Wuhan, China) and cultured in Ham’s F-12K (Procell, Wuhan, China) containing 10% fetal bovine serum (BI, Beit Haemek, Israel) and 1% penicillin–streptomycin solution (Gibco, Waltham, MA, USA). Primary rat lung fibroblasts were obtained after the rats were euthanized. The lung tissues were excised and spread flat in a culture flask. The fibroblasts were cultured in DMEM–high glucose (Gibco, Waltham, MA, USA) containing 20% fetal bovine serum (BI, Beit Haemek, Israel) and 1% penicillin–streptomycin solution (Gibco, Waltham, MA, USA), and the third to seventh generations of the primary rat lung fibroblasts were used for experiments. All cells were incubated with bergenin (with or without 10 ng/mL TGF-β1) (PeproTech, Rocky Hill, NJ, USA) according to different requirements.

### 2.3. Histological Analysis

The excised tissue was fixed with 4% paraformaldehyde, embedded in paraffin, and sectioned. The sections were stained with hematoxylin and eosin (HE). The Ashcroft score is a simple method of estimating the severity of pulmonary fibrosis on a numerical scale. Lung paraffin sections stained with hematoxylin and eosin were systematically scanned in a microscope using a ×10 objective. The severity of interstitial fibrosis was assessed individually for each consecutive region and assigned a score between 0 and 8 using a predetermined severity. Each tissue section was scored by three independent investigators, and the average score of the three investigators was used as the fibrosis score for that section. For the details of this approach, refer to the study by Ashcroft et al. [38].

### 2.4. Immunohistochemistry

Tissue sections were deparaffinized with xylene and dehydrated with ethanol. The antigen was extracted using microwaves. The sample was incubated with goat serum and then incubated overnight with type I collagen polyclonal antibody (1:200, Millipore, Burlington, VT, USA) or α-SMA monoclonal antibody (1:500, CST, Danvers, MA, USA) at 4 °C. The cells were incubated for 1 h at room temperature with goat anti-rabbit immunoglobulin G monoclonal antibody (1:5000, SAB, Nanjing, China). The sections were stained and rinsed with phosphate-buffered saline (PBS) after incubation with 0.05% diaminobenzidine. 

### 2.5. Hydroxyproline Assay

Hydroxyproline levels in the lung tissue were quantified using hydroxyproline detection kits (Nanjing Jiancheng Biotechnology Institute, Nanjing, China) according to the manufacturer’s instructions.

### 2.6. Measurement of Superoxide Dismutase, Malondialdehyde, and Glutathione

Malondialdehyde (MDA), glutathione (GSH), and superoxide dismutase (SOD) detection kits (Nanjing Jiancheng Institute of Biotechnology, Nanjing, China) were used to determine the activity of total SOD and the contents of MDA and GSH.

GSH: Reduced glutathione (GSH) can react with 2-nitrobenzoic acid (DTNB) to form a yellow compound that can be quantified at 405 nm. The lung tissue of mice was weighed and constituted into 10% tissue homogenate with saline, then the homogenate was centrifuged, and the supernatant was collected. The precipitating agent in the kit was added, and the supernatant was collected by centrifugation, and then the chromogenic reagent and buffer in the kit were added in turn. The absorbance value of each well was measured at 405 nm with a microplate reader after mixing.

MDA: Malondialdehyde (MDA) in the degradation product of lipid peroxide can be combined with thiobarbituric acid (TBA) to form a red product with a maximum absorption peak at 532 nm. The lung tissue of mice was weighed and constituted into 10% tissue homogenate with saline. The ethanol and the clarifying agent in the kit were successively added and mixed. Then the buffer, chromogenic agent, and 50% glacial acetic acid in the kit were successively added and bathed in water at 95 °C for 40 min. After centrifugation, the supernatant was collected to measure the absorbance with a microplate reader at 532 nm.

Total SOD: Wst-1 can react with superoxide anion (O_2_^.−^) catalyzed by xanthine oxidase (XO) to produce water-soluble formazan dye. As SOD can catalyze the disproportionation of superoxide anion, the reaction can be inhibited by SOD. Therefore, the activity of SOD is negatively correlated with the generation of formazan dye, and the activity of SOD can be calculated by the colorimetric analysis of the WST-1 product. The lung tissue of mice was weighed and constituted into 10% tissue homogenate with saline. After centrifugation, the supernatant was collected, and the protein concentration was determined by the BCA method. Distilled water, enzyme application solution, enzyme diluent, and substrate application solution from the kit were added to the supernatant successively. The mixture was incubated at 37 °C for 20 min, and the absorbance was measured at 450 nm with a microplate reader.

### 2.7. ROS Staining of Lung Tissue

Frozen sections of the lung tissue were warmed to room temperature, and the residual moisture was dried. A histochemical pen was used to draw a circle around the tissue to prevent the antibody from diffusing, and an autofluorescence quencher was added to the circle. The samples were rinsed with running water. The ROS dye solution was added dropwise to the circle, and the samples were incubated at 37 °C, in a dark incubator, for 30 min. The slides were then placed in PBS (pH 7.4) and washed using a decolorizing shaker. After the sections were dried slightly, the nuclei were counterstained by adding the DAPI dye solution to the circle and incubating at room temperature in the dark. The slides were placed in PBS (pH 7.4) and washed using a decolorizing shaker. After the slices were dried slightly, they were mounted on the anti-fluorescence quenching mounting tablets, and the slices were placed under a fluorescence microscope to observe and collect images. 

### 2.8. RNA Extraction and Quantitative Real-Time Polymerase Chain Reaction

RNA was extracted from the cells and lung tissues using TRIzol (Thermo Fisher Scientific, Waltham, MA, USA). A reverse transcription kit (Thermo Fisher Scientific, Waltham, MA, USA) was used to reverse transcribe cDNA. Bio-Rad CFX96 Touch Real-time PCR Detection System (Bio-Rad, Hercules, CA, USA) and SYBR Green (Promega, Madison, WI, USA) were used for real-time quantitative PCR. The conditions were as follows: 95 °C, 2 min; 95 °C, 3 s; 60 °C, 30 s; 40 cycles; the melting curve was found to be in the range 60–95 °C. The primer sequences are shown in Table 1.

### 2.9. Western Blotting 

A radioimmunoprecipitation (RIPA) lysate was used to extract the total protein from the lung tissue and cells. The protein concentration was determined using the bisphenolic acid (BCA) protocol. The proteins were subjected to 10% sodium lauryl sulfate–polyacrylamide gel electrophoresis, and the proteins were transferred to a PVDF membrane (Bio-Rad, USA), blocked with 5% (*w*/*v*) skimmed milk containing 0.1% TBS buffer Tween 20 (*v*/*v*). The membranes were exposed to α-SMA monoclonal antibody (1:1000, CST, Danvers, MA, USA), collagen I polyclonal antibody (1:1000, Millipore, Burlington, VT, USA), GAPDH monoclonal antibody (1:5000, SAB, Nanjing, China), HO-1 monoclonal antibody (1:2000, Abcam, Cambridge, UK), NQO1 monoclonal antibody (1:10,000, Abcam, Cambridge, UK), Nrf2 monoclonal antibody (1:1000, Abcam, Cambridge, UK), p62 monoclonal antibody (1:10,000, Abcam, Cambridge, UK), and phosphorylated p62 S349 monoclonal antibody (1:1000, Abcam, Cambridge, UK) at 4 °C, overnight. Each membrane was incubated with goat anti-rabbit immunoglobulin G monoclonal antibody (1:5000, SAB, Nanjing, China) labeled with horseradish peroxidase for 2 h after washing thrice with TBST at room temperature, and Luminata™ Crescendo chemiluminescent horseradish peroxidase substrate (Millipore, Burlington, VT, USA) was used to observe the bands. A GeneGnome XRQ Imager (Syngene, Cambridge, UK) was used for scanning.

### 2.10. Cell Viability Assay

The cell viability was measured using Cell Counting Kit-8 (CKK-8) (Biosharp, Hefei, China) according to the manufacturer’s instructions. The cells were seeded in 96-well plates at a density of 4000 cells per well and exposed to different concentrations of compounds for the indicated times. A 10 μL volume of working reagent was added to each well, and the wells were incubated at 37 °C. Absorbance was measured at 450 nm. The optical density is directly proportional to the number of living cells on the photosensitive plate.

### 2.11. Immunofluorescence Assays

Immunofluorescent signals were used to identify vimentin expression in rat primary lung fibroblasts. Ice-cold PBS was used to wash the cells, and the cells were fixed with 4% paraformaldehyde and permeated with 0.5% (*v/v*) Triton X-100. The samples were incubated with goat serum blocker (ZSGB Bio, Beijing, China) and then incubated overnight with polyclonal antibodies against vimentin (1:300, Abcam, Cambridge, UK) at 4 °C. After washing, the cells were incubated with fluorescent secondary antibody (1:1000, SAB, Nanjing, China) in PBS containing 0.1% (*v/v*) Tween 20 at room temperature for 1 h and stained with DAPI. The cells were observed under a fluorescent microscope. 

### 2.12. Cellular ROS Detection

Following different treatments, the NIH3T3 cells were incubated with the ROS fluorescent probe H2DCFDA (Beyotime Biotechnology, Shanghai, China) at 37 °C for 30 min, washed with DMEM, and then observed using a fluorescence microscope (Nikon Ti-s, Tokyo, Japan). The contents of ROS were detected using flow cytometry (BD LSRFortessa, Franklin Lakes, NJ, USA).

### 2.13. siRNA Transfection

According to the manufacturer’s instructions. The cells were transfected with siRNA targeting Nrf2, siRNA targeting p62, or control siRNA (Santa Cruz, Santa Cruz, CA, USA) using Lipofectamine 2000 (Invitrogen, Waltham, MA, USA).

### 2.14. Determination of Respiratory Function

After the mice were anesthetized, the trachea was intubated, and respiratory function indices such as respiratory rate, airway resistance, lung compliance, and pulmonary ventilation of the mice were measured using the BUXCO mouse respiratory function detection system (Max II, Wilmington, NC, USA). 

### 2.15. Statistical Analysis

All data were analyzed using GraphPad Prism 8.3.1 (San Diego, CA, USA). A *t*-test was used to compare the two groups, one-way analysis of variance and Tukey’s multiple comparisons test were used for the comparison between the groups, and the log-rank test was used for survival analysis. Statistical significance was set at *p* < 0.05.

## 3. Results

### 3.1. Bergenin Attenuated Bleomycin-Induced Pulmonary Fibrosis in Mice

**Figure 1:**
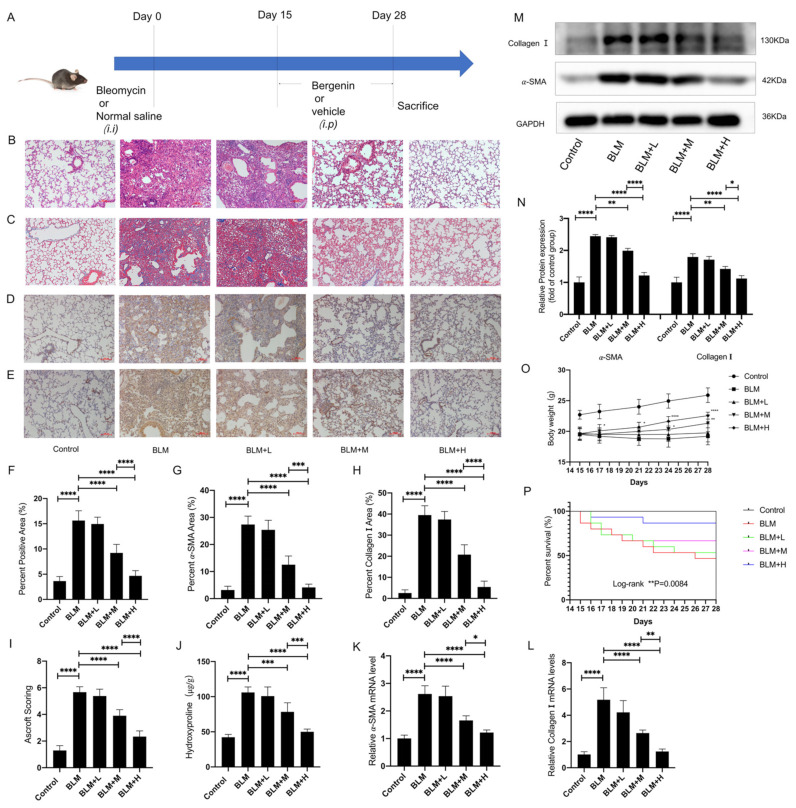
Bergenin inhibits pulmonary fibrosis in mice. On days 15 to 28, after intratracheal injection of bleomycin, intraperitoneal injec-tions of bergenin were administered at high (25 mg/kg), moderate (5 mg/kg), and low (1 mg/kg) doses to evaluate its therapeutic effects on pulmonary fibrosis (**A**). HE staining and Masson staining were used to evaluate the lung morphology and ECM deposition (**B**,**C**,**F**). The fibrosis was scored using Ashcroft scoring (**I**), and immunohistochemistry was used to detect the positive expression of α-SMA and type I collagen in the lung tissue (**D**,**E**,**G**,**H**) (magnification ×100). The total hy-droxyproline content of the lung tissue was determined using biochemical methods (**J**). Western blotting and quantitative polymerase chain reaction (Q-PCR) were used to detect the protein and mRNA expression levels of α-SMA and type I col-lagen (**K**–**N**). Changes in body weight and survival numbers of mice in each group were recorded daily (**O**,**P**). The following notations are used: Control for the control group; BLM for the bleomycin group; BLM + L for the bleomycin + 1 mg/kg bergenin group; BLM + M for the bleomycin + 5 mg/kg bergenin group; and BLM + H for the bleomycin + 25 mg/kg bergenin group. Data are expressed as mean ± SD, n = 6–8, * *p* < 0.05; ** *p* < 0.01; *** *p* < 0.001; **** *p* < 0.0001.

Inflammation was the main pathological feature in the bleomycin model from the 1st to the 14th day, and fibrosis gradually worsened from the 15th to the 28th day [39,40]. Therefore, we chose to intervene on days 15–28 to observe the direct antifibrosis effect of bergenin. We designed a plan to determine whether bergenin exerted direct antifibrotic effects (Figure 1A). Hematoxylin and eosin (HE) and Masson staining showed that the moderate and high doses of bergenin significantly reduced the accumulation of ECM and the thickness of the alveolar wall (Figure 1B,C,F). The same conclusion was reached upon analyzing the Ashcroft score (Figure 1I). Immunohistochemical data showed that bergenin attenuated the increase in α-SMA and type I collagen–positive areas induced by bleomycin (Figure 1D,E,G,H). Accordingly, high and moderate doses of bergenin significantly decreased the mRNA and protein expression levels of α-SMA and type I collagen compared to those in the group treated with bleomycin alone (Figure 1K–N). The antifibrosis effect of bergenin was further confirmed by the reduction of the hydroxyproline content (Figure 1J). Changes in body weight and survival curves of each group from days 15 to 28 revealed that moderate and high doses of bergenin can effectively improve weight loss and reduce the number of deaths caused by bleomycin (Figure 1O,P). In the above results, the effect of high-dose bergenin is more obvious than that of the middle dose. These results indicated that bergenin could effectively inhibit pulmonary fibrosis in mice induced by bleomycin.

### 3.2. Bergenin Activated Nrf2 and Attenuated Oxidative Stress in Bleomycin-Treated Mice

**Figure 2:**
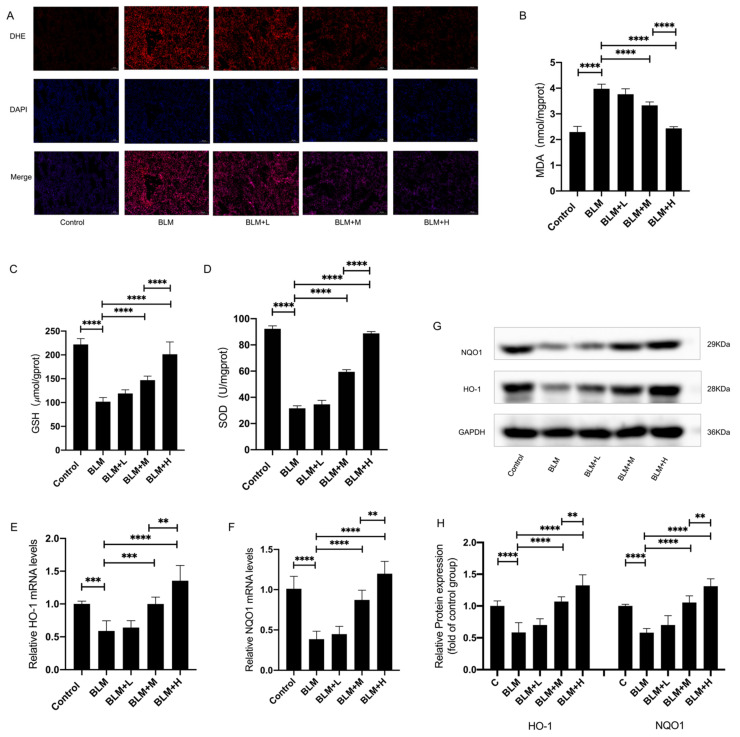
Bergenin exerts its antioxidant effect in bleomycin-treated mice. On days 15 to 28, after intratracheal injection of bleomycin, intraperitoneal injections of bergenin were administered at high (25 mg/kg), moderate (5 mg/kg), and low (1 mg/kg) doses. DHE staining was used to compare the level of ROS in the lung tissues of each group (A) (magnification ×100). The MDA and GSH levels and the activity of SOD were measured following the manufacturer’s protocol (**B**–**D**). Q-PCR and Western blotting were used to detect the mRNA and protein expression levels of HO-1 and NQO1 (**E**–**H**). The following notations are used: Control for the control group; BLM for the bleomycin group; BLM + L for the bleomycin + 1 mg/kg bergenin group; BLM + M for the bleomycin + 5 mg/kg bergenin group; and BLM + H for the bleomycin + 25 mg/kg bergenin group. Data are expressed as mean ± SD, *n* = 6–8, ** *p* < 0.01; *** *p* < 0.001; **** *p* < 0.0001.

We investigated whether the inhibitory effect of bergenin on pulmonary fibrosis is related to its antioxidant activity while taking the important role of oxidative stress in the occurrence and development of pulmonary fibrosis and the strong antioxidant activity of bergenin into account. We measured the levels of ROS (Figure 2A), malondialdehyde (MDA), and glutathione (GSH) and the activity of superoxide dismutase (SOD) in the lung tissue to estimate the degree of oxidative damage (Figure 2B–D) and found that moderate and high doses of bergenin decreased ROS levels in the lung tissues. The moderate and high doses of bergenin effectively increased the levels of GSH and the activity of SOD, and conversely, effectively decreased the levels of MDA. In addition, we found that moderate and high doses of bergenin can effectively increase the mRNA and protein expression levels of the antioxidant genes HO-1 and NQO1 (Figure 2E–H). Among these effects, the effect of high-dose bergenin was more significant than that of the moderate dose. These results indicated that bergenin exerted a protective effect against oxidative stress caused by bleomycin. Furthermore, this mechanism of action may be related to bergenin activating Nrf2 and increasing the level of antioxidant enzymes in the body.

### 3.3. Bergenin Inhibited the TGF-β1-Induced Differentiation of Lung Fibroblasts into Myofibroblasts 

**Figure 3:**
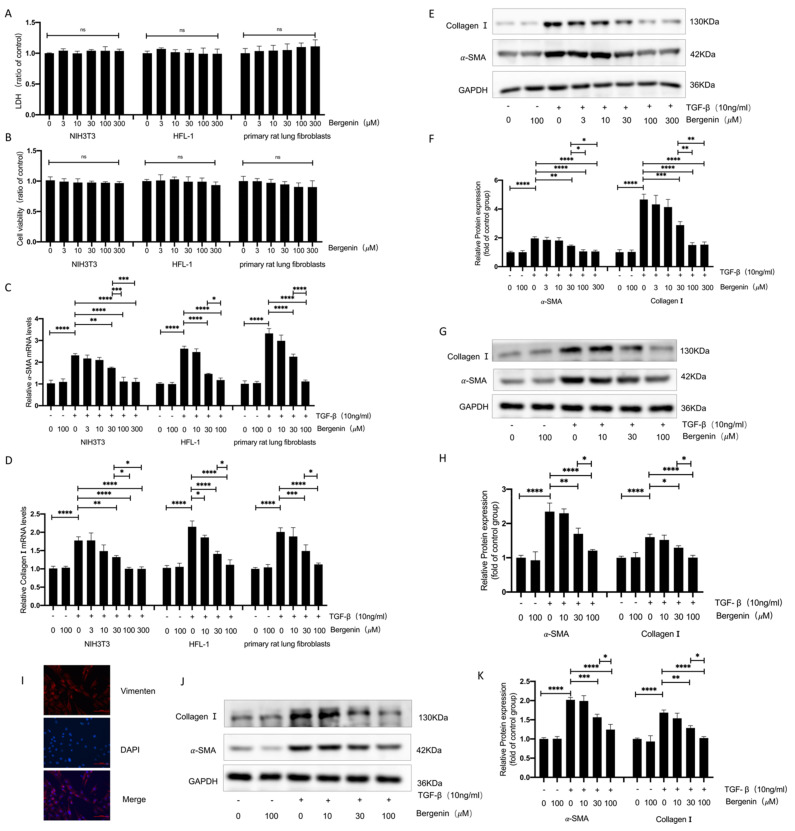
Bergenin can reduce the differentiation of myofibroblasts induced by TGF-β1 in lung fibroblasts. The NIH3T3 cells, HFL-1 cells, and primary rat lung fibroblasts were incubated with different doses of bergenin and TGF-β1 (10 ng/mL) for 24 h. The effects of the different concentrations of bergenin on cell viability were evaluated, and the LDH activity of the cell supernatant was measured following the manufacturer’s protocol (**A**,**B**). Q-PCR was used to detect the levels of mRNA expression in α-SMA and type I collagen (**C**,**D**). The protein expression levels of α-SMA and type I collagen were detected by Western blotting (**E**–**H**,**J**–**K**). The primary rat lung fibroblasts were validated with a vimentin immunofluorescence experiment (**I**). Data are expressed as mean ± SD, and all experiments were repeated independently at least three times, * *p* < 0.05; ** *p* < 0.01; *** *p* < 0.001; **** *p* < 0.0001.

In order to further study the antifibrosis mechanism of bergenin, we tested the effect of bergenin on TGF-β1 (10 ng/mL)-induced myofibroblast differentiation. Initially, we analyzed the effects of five bergenin concentrations (3, 10, 30, 100, and 300 μM) on cell supernatant lactate dehydrogenase (LDH) activity and cell viability of NIH3T3 cells and found that these parameters were unaffected by them (Figure 3A,B). Therefore, we selected these five concentrations for follow-up experiments. TGF-β1 significantly upregulated the mRNA and protein expression levels of α-SMA and type I collagen. The mRNA and protein expression levels of α-SMA and type I collagen were significantly reduced by 30, 100, and 300 μM of bergenin, with 100 μM of bergenin showing a more significant effect on α-SMA and type I collagen than 30 μM of bergenin did. However, there was no significant difference between the effects of 300 and 100 μM of bergenin (Figure 3C–F). These results prove that bergenin can effectively inhibit the differentiation of fibroblasts into myofibroblasts in vitro. We also verified this conclusion in HFL-1 cells (Figure 3A–D,G,H) and rat primary lung fibroblasts (Figure 3A–D,I–K).

### 3.4. The Inhibitory Effect of Bergenin on Myofibroblast Differentiation Depends on the Activation of Nrf2

**Figure 4:**
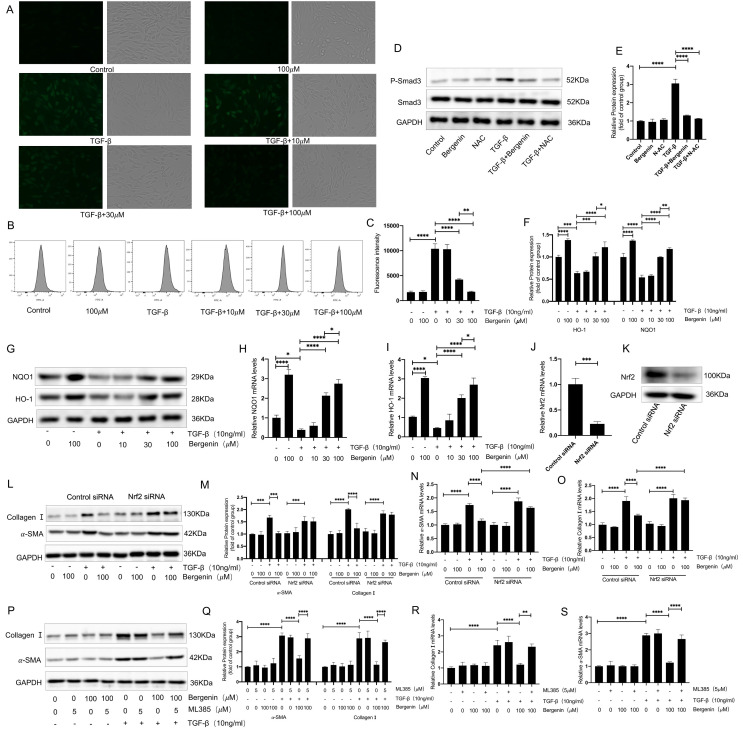
Bergenin decreases the level of ROS and inhibits myofibroblast transformation by activating Nrf2. Bergenin with or without TGF-β1 (10 ng/mL) was incubated with NIH3T3 cells treated in different ways. NIH3T3 cells were incubated with three different concentrations of bergenin (10, 30, and 100 μM) and TGF-β1 (10 ng/mL) for 24 h, followed by incubation with the ROS fluorescent probe DCFH-DA. The cells were then observed under a fluorescence microscope, and the content of ROS was detected by flow cytometry (**A**–**C**). Western blotting was used to compare the effect of 100 μM of bergenin and 5 mM of NAC on Smad3 phosphorylation induced by TGF-β1 (**D**,**E**). Q-PCR and Western blotting were used to evaluate the mRNA and protein expression levels of HO-1 and NQO1 (**F**–**I**). ML385 (5 μM) was added to the medium and incubated for 1 h before NIH3T3 cells were incubated with bergenin and TGF-β1 (10 ng/mL). Q-PCR and Western blotting were used to detect the mRNA and protein expression levels of type I collagen and α-SMA (**P**–**S**). After transfection with Nrf2-targeted siRNA or control siRNA, Q-PCR and Western blotting were used to detect the mRNA and protein expression levels of Nrf2 (**J**,**K**). Q-PCR and Western blotting were also used to detect the mRNA and protein expression levels of type I collagen and α-SMA (**L**–**O**). Data are expressed as mean ± SD, and all experiments were repeated independently at least three times, * *p* < 0.05; ** *p* < 0.01; *** *p* < 0.001; **** *p* < 0.0001.

In vivo experiments revealed that bergenin could effectively increase the levels of multiple antioxidant indicators in lung tissues and effectively suppress oxidative stress. Therefore, we chose to evaluate the changes of the Nrf2 pathway related to oxidative stress for subsequent in vitro experiments, and NIH3T3 cells were selected for subsequent experiments. We selected three concentrations of bergenin (10, 30, and 100 μΜ) for the follow-up studies, based on the results of our study on the inhibitory effect of bergenin on myofibroblast transformation in NIH3T3 cells. Bergenin was added during the TGF-β1-induced myofibroblast differentiation process in the NIH3T3 cells (10 ng/mL). By using fluorescence microscopy and flow cytometry, we found that 30 and 100 μΜ of bergenin could effectively reduce the ROS levels and that the decrease of ROS was more pronounced in the 100 μΜ group (Figure 4A–C). Given that ROS plays an important role in the activation of the TGF-β/Smad signaling pathway, we compared the effects of 100 μM of bergenin and 5 mM of NAC on the phosphorylation of Smad3, and the results showed that both can reduce the increase in Smad3 phosphorylation caused by TGF-β1 (Figure 4D,E). It suggested that the inhibitory effect of bergenin on Smad3 phosphorylation may be related to its powerful antioxidant activity. To further explore the underlying mechanism for the decrease in ROS caused by bergenin, we observed the changes in the expression levels of HO-1 and NQO1 during the process of myofibroblast differentiation in the NIH3T3 cells induced by TGF-β1 (10 ng/mL). We found that 30 and 100 μM of bergenin effectively increased the mRNA and protein expression levels of HO-1 and NQO1 and that 100 μM of bergenin caused a higher increase than that caused by a 30 μM dose (Figure 4F–I). It indicated that bergenin can specifically activate the Nrf2 signaling pathway, thereby exerting its antioxidant effect. To clarify the role of Nrf2 in bergenin’s inhibition of myofibroblast transformation, we transfected small-interfering RNA (siRNA) targeting Nrf2 or incubated the Nrf2-inhibitor ML385 with bergenin to investigate the effect of Nrf2 on the transformation of the NIH3T3 cells into myofibroblasts by bergenin. Our results showed that ML385 could inhibit the decrease in the expression levels of mRNA and protein in α-SMA and type I collagen caused by bergenin (Figure 4P–S). Compared to the effects of ML385, transfection with Nrf2 siRNA produced a similar effect (Figure 4J–O). It showed that bergenin can inhibit TGF-β1-induced fibroblast to myofibroblast transformation by promoting the activation of Nrf2.

### 3.5. The p62–Nrf2 Regulatory Loop Mediates the Antioxidation and Antifibrosis Effect of Bergenin

**Figure 5:**
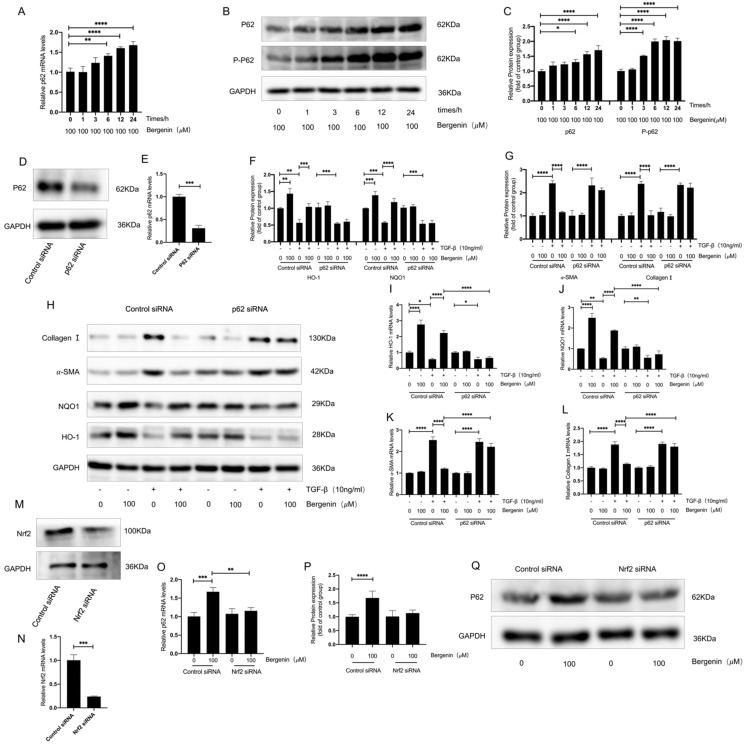
Bergenin activates Nrf2 and inhibits the transformation of myofibroblasts by forming a positive feedback loop between Nrf2 and p62. NIH3T3 cells were used to investigate the effect of bergenin on p62 and the relationship between the effect of bergenin on p62 and Nrf2. NIH3T3 cells were exposed to 100 μM of bergenin for 0, 1, 3, 6, 12, and 24 h, and the expression levels of p62 and P-p62 were detected using Q-PCR and Western blotting (**A**–**C**). After transfection with control siRNA or p62-targeted siRNA, the mRNA and protein expression levels of p62 were detected by Q-PCR and Western blotting (**D**,**E**). Q-PCR and Western blotting were also used to detect the mRNA and protein expression levels of type I collagen, α-SMA, HO-1, and NQO1 (**F**–**L**). After transfection with Nrf2-targeted siRNA or control siRNA (**M**,**N**), NIH3T3 cells were incubated with 100 μM of bergenin for 24 h, and the mRNA and protein levels of p62 were detected by Q-PCR and Western blotting (**O**–**Q**). Data are expressed as mean ± SD, and all experiments were repeated independently at least three times, * *p* < 0.05; ** *p* < 0.01; *** *p* < 0.001; **** *p* < 0.0001.

We further explored the mechanism by which bergenin activated Nrf2 and found that bergenin promoted the protein expression levels of p62/SQSTM1 (p62) and increased the phosphorylated-p62 (P-p62) levels (Figure 5A–C). In normal cells, p62 interacts with the Nrf2 pathway, and once p62 is phosphorylated, Nrf2 is activated. When we specifically silenced the expression of p62, the effect of bergenin on the mRNA and the protein expression levels of type I collagen, α-SMA, HO-1, and NQO1 after TGF-β1 induction were inhibited (Figure 5D–L). This suggested that p62 mediates the activation of bergenin on Nrf2. Studies have shown that the activation of Nrf2 can upregulate the expression of p62, thus forming a positive feedback loop between Nrf2 and p62 [41,42]. In short, P-p62 induces Nrf2 activation, and Nrf2 activation further leads to an increase in p62 expression. When we specifically silenced the expression of Nrf2, we found that the effect of bergenin on p62 was inhibited (Figure 5M–Q). These results indicate that the activation of Nrf2 by bergenin may be mediated by the increased phosphorylation of p62 and that bergenin activates a positive feedback loop between p62 and Nrf2. Therefore, we have reason to believe that bergenin exerts its antifibrosis and antioxidant effects by forming a positive feedback loop between Nrf2 and p62.

### 3.6. Nrf2 Mediated the Anti-pulmonary Fibrosis Effects of Bergenin In Vivo

**Figure 6:**
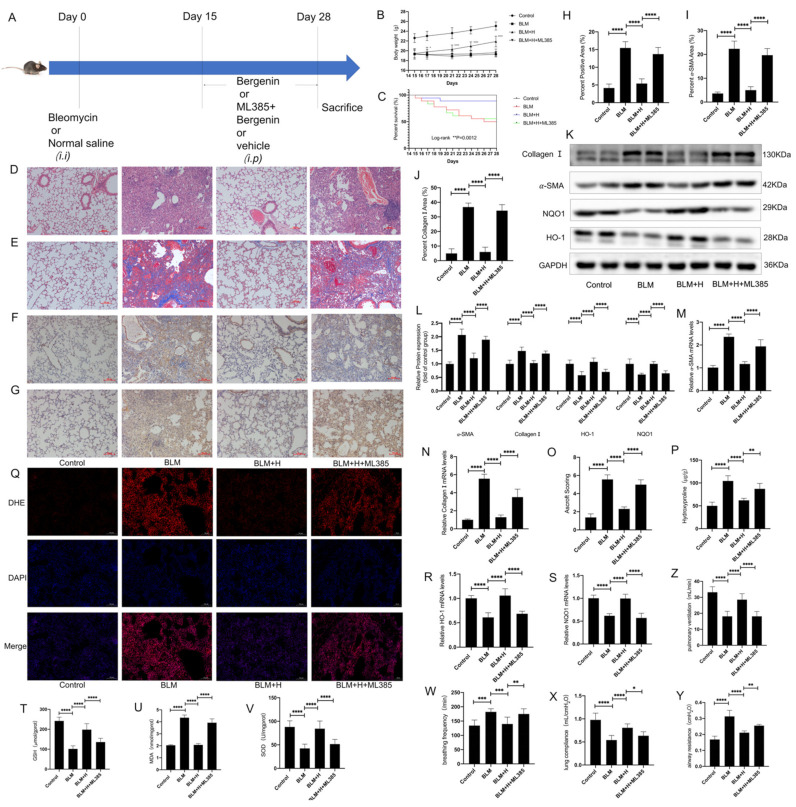
The Nrf2 inhibitor ML385 inhibited the therapeutic effect of bergenin on pulmonary fibrosis in mice. An intraperitoneal injection of 30 mg/kg of ML385 was administered 3 h before the intraperitoneal injection of high-dose bergenin (25 mg/kg) and compared with the mice with pulmonary fibrosis and the mice treated with high-dose bergenin (**A**). Changes in body weight and survival numbers of mice in each group were recorded daily (**B**,**C**). HE staining and Masson staining were used to evaluate the lung morphology and ECM deposition (magnification ×100) (**D**,**E**,**H**). Fibrosis was evaluated using the Ashcroft score (**O**). Immunohistochemistry was used to detect positive expression of type I collagen and α-SMA in lung tissue (magnification ×100) (**F**,**G**,**I**,**J**). Q-PCR was used to detect the mRNA expression levels of type I collagen, α-SMA, HO-1, and NQO1 (**M**,**N**,**R**,**S**), and Western blotting was used to detect the protein expression levels of type I collagen, α-SMA, HO-1, and NQO1 (**K**,**L**). The total hydroxyproline contents of the lung tissues were determined using biochemical methods (**P**). The levels of GSH and MDA and the activity of SOD were measured following the manufacturer’s protocol (**T**–**V**). DHE staining was used to compare the differences in ROS content in the lung tissues of each group (magnification ×100) (**Q**). The differences in respiratory rate, airway resistance, lung compliance, and pulmonary ventilation between groups were determined using the BUXCO system (**W**–**Z**). The following notations are used: Control for control; BLM for bleomycin; BLM + H for bleomycin + 25 mg/kg bergenin; BLM + H + ML385 for bleomycin + 30 mg/kg ML385 + 25 mg/kg bergenin. Data are expressed as mean ± SD, *n* = 8–9, * *p* < 0.05; ** *p* < 0.01; *** *p* < 0.001; **** *p* < 0.0001.

According to the results of our in vitro experiments, in order to further verify whether bergenin can reduce oxidative stress and pulmonary fibrosis by activating Nrf2, we used 30 mg/kg of ML385 as a pretreatment and administered it intraperitoneally 3 h before bergenin treatment (Figure 6A). ML385 inhibited the effects of bergenin on body weight and survival rate in mice with pulmonary fibrosis (Figure 6B,C). The effects of bergenin on lung tissue morphology and alveolar wall thickness were inhibited by ML385 according to the results of HE staining and Masson staining (Figure 6D,E,H), which was also confirmed by the results of Ashcroft score and hydroxyproline (Figure 6O,P). Immunohistochemistry revealed that the inhibitory effects of bergenin on the expression of α-SMA and type I collagen were also inhibited by ML385 (Figure 6F,G,I,J), and the result was consistent with the results of Western blotting and Q-PCR (Figure 6K–N). These results indicated that the antifibrotic effect of bergenin could be inhibited by ML385. Similarly, we found that ML385 inhibited the increase in mRNA and protein expression levels of HO-1 and NQO1 caused by bergenin (Figure 6K,L,R,S). ML385 simultaneously influenced the levels of MDA and GSH and the activity of SOD in lung tissues (Figure 6T–V). In addition, we used DHE staining to determine whether ML385 can interfere with the attenuating effect of bergenin on ROS in lung tissue (Figure 6Q). These results indicated that the antioxidant effect of bergenin could also be inhibited by ML385. To explore the effects of bergenin on lung function in mice, we determined that bergenin could effectively improve airway resistance, lung compliance, and pulmonary ventilation and reduce the respiratory rate of mice with bleomycin-induced pulmonary fibrosis through the BUXCO system, and these effects can also be inhibited by ML385 (Figure 6W–Z). These results strongly suggested that Nrf2 plays a central role in bringing about the antifibrotic and antioxidative stress effects of bergenin. 

### 3.7. Schematic of a Model of the Anti-pulmonary Fibrosis Effect of Bergenin

**Figure 7:**
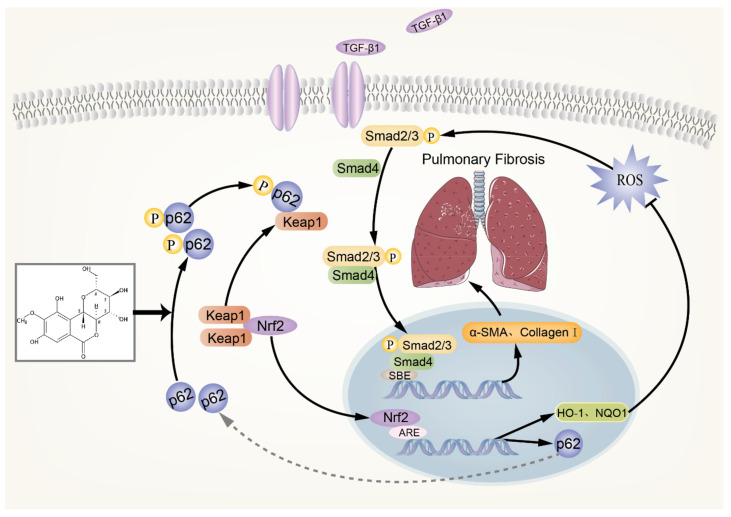
Schematic of a model of the anti-pulmonary fibrosis effect of bergenin.

In summary (Figure 7), bergenin increases the phosphorylation level of p62, with the phosphorylated p62 binding to Keap1, thereby promoting the nuclear translocation of Nrf2 and increasing the expression of HO-1, NQO1, and other downstream antioxidant genes of Nrf2, as well as clearing the excessive ROS in the cells. Thus, the TGF-β/Smad signaling pathway is inhibited, and the expression levels of α-SMA, type I collagen, and other profibrotic genes are decreased. In addition, Nrf2 activation can promote the expression of p62, resulting in a positive feedback effect of p62–Nrf2 and amplifying the anti-pulmonary fibrosis effect of bergenin.

## 4. Discussion

For the first time, we observed that bergenin exerts its powerful antioxidant effect and inhibits TGF-β1-induced myofibroblast transformation through a p62–Nrf2 positive feedback loop, thereby alleviating bleomycin-induced pulmonary fibrosis.

The pulmonary fibrosis model constructed by intratracheal injection of bleomycin is one of the most important tools for studying the pathological mechanism of IPF and finding new compounds to treat IPF. The mice generally undergo the following stages after intratracheal injection of bleomycin: On days 1 to 7, acute lung injury and inflammation occur, and the epithelium is extensively damaged; inflammatory cells increase, and a variety of inflammatory mediators are activated. From day 7 to 14, the inflammation gradually subsides. However, the inflammatory response remains the main pathological feature of this stage. After day 14, the myofibroblast population and ECM deposition increases, and collagen and other ECM depositions reach their peak on day 28 [39]. Several studies have started drug intervention from day 1 to day 14 after modeling [40]. However, the influence of the anti-inflammatory effect of drugs on the treatment of pulmonary fibrosis cannot be ruled out. In our study, different concentrations of bergenin were intraperitoneally injected from day 15 to day 28 to study whether bergenin has a direct anti-pulmonary fibrotic effect, and the results showed that bergenin could directly inhibit pulmonary fibrosis.

It is reported that oxidative stress plays an important role in the occurrence and development of pulmonary fibrosis [7]. Oxidative stress can promote the formation of myofibroblasts by promoting the secretion of a variety of cytokines such as TGF-β. The increase in the number of myofibroblasts caused by oxidative stress can lead to the increase of ECM, which further promotes the development of fibrosis [8]. It has been recognized that pulmonary fibrosis can be treated via antioxidant effects. For example, N-acetylcysteine has been shown in many studies to reduce oxidative stress and inhibit the transformation of myofibroblasts in vitro and to reduce pulmonary fibrosis in vivo, due to its antioxidant effect [43]. Bergenin has strong antioxidant effects. To study whether bergenin has a direct inhibitory effect on the occurrence and development of lung fibrosis by interfering with oxidant stress, it was intraperitoneally injected into mice with bleomycin-induced pulmonary fibrosis. The results showed that bergenin reduced the levels of ROS and MDA and increased the levels of GSH, the activity of SOD, and the expression levels of HO-1 and NQO1, suggesting that the anti-pulmonary fibrosis effect of bergenin is related to its antioxidant effect. In the process of transforming TGF-induced fibroblasts into myofibroblasts, myofibroblast transformation can be inhibited by reducing the level of ROS [23,24]. Based on the in vivo studies, we found that bergenin can also reduce the phosphorylation level of Smad3 and the expression levels of type I collagen and α-SMA by reducing the level of ROS during the transformation of myofibroblasts. In addition, myofibroblasts are an important source of ROS in fibrotic lung tissue, excessive levels of ROS are released from myofibroblasts that can cause apoptosis of the alveolar epithelial cells, thus aggravating pulmonary fibrosis [44]. Our study proved that bergenin can reduce the level of ROS, which may reduce the apoptosis of epithelial cells, and this may be one of the mechanisms of bergenin in the treatment of pulmonary fibrosis.

Many substances in the body play a role in antioxidant defense in preventing damage caused by oxidative stress. Among these, Nrf2 is considered an important transcription factor in the antioxidant response [45]. Many Nrf2 activators, such as sulforaphane, have been demonstrated to effectively reduce the ROS levels and reduce fibrosis by activating Nrf2 [46,47,48,49]. We first found that bergenin can activate Nrf2 and promote the expression of HO-1 and NQO1 on NIH3T3 cells. To investigate whether Nrf2 mediates the antifibrosis effect of bergenin, we silenced Nrf2 and found that bergenin’s effect on myofibroblast transformation was inhibited. Thus, we believe that Nrf2 mediates the antioxidative stress and antifibrotic effects of bergenin. 

In normal cells, p62 interacts with the Nrf2 pathway. Once p62 is phosphorylated, it physically interacts with the Nrf2 inhibitory protein Keap1. Subsequently, Nrf2 dissociates from Keap1 and transfers to the nucleus, and the transcriptional activity of Nrf2 is enhanced to promote the cytoprotective gene expression [41,42]. Bergenin activates Nrf2 by promoting the phosphorylation of p62 along with causing an increase in the level of p62. However, after silencing Nrf2, the promotion of bergenin on the expression of p62 was inhibited. Therefore, our research showed, for the first time, that bergenin creates a positive feedback loop between p62 and Nrf2. According to the research, proteases such as KHK-A and mTORC1 can regulate the phosphorylation of p62, and high expression of the p62 protein can also enhance the phosphorylation level of p62 [50,51]. Our study showed that bergenin can increase the phosphorylation level of p62, but further research is needed to clarify its mechanism. It has been proven that p62 plays an important role in inhibiting the occurrence and development of fibrosis [52]. However, the specific mechanism is still unclear. Combined with our research results, we have reason to believe that the p62–Nrf2 regulatory loop may be a new drug target for the treatment of pulmonary fibrosis.

In recent years, studies have reported that PPARγ mediates certain pharmacological effects of bergenin, but its detailed mechanism is not clear [53,54]. It was discovered that PPARγ and Nrf2 overlap in the protection of mitochondria, defense against oxidative stress, and other functions [55], and the activation of PPARγ is affected by Nrf2 [56]. Given that PPARγ also plays an important role in inhibiting the process of pulmonary fibrosis, whether the antifibrotic effect of bergenin mediated by Nrf2 is related to the activation of PPARγ in addition to the activation of the body’s antioxidant system remains to be further studied. According to our findings, bergenin exerts a direct anti-pulmonary fibrosis effect, which is mediated by activating the p62–Nrf2 positive feedback loop to inhibit oxidative stress. Therefore, our study, for the first time, proposed that the p62–Nrf2 regulatory loop is a new drug target for the treatment of pulmonary fibrosis, which has a definite theoretical and clinical application value.

## 5. Conclusions

By observing the relationship between Nrf2 and p62, we clarified how bergenin exerted its antifibrotic effects. Bergenin increases the phosphorylation and expression of p62, promoting the activation of Nrf2, which inhibits the TGF-β/Smad signaling pathway. Therefore, the expression of α-SMA, type I collagen, and other profibrotic genes is decreased. In addition, the activation of Nrf2 can promote the expression of p62, thus forming a positive feedback loop between p62 and Nrf2, amplifying the anti-pulmonary fibrosis effect of bergenin. Our findings suggested that bergenin could be a therapeutic agent for pulmonary fibrosis treatment, and we propose that the p62–Nrf2 regulatory loop may be a potential therapeutic target for pulmonary fibrosis.

## Figures and Tables

**Table 1:** The primer sequence.

	Forward	Reverse
Mouse GAPDH (NM_001289726)	5′-GGTTGTCTCCTGCGACTTCA-3′	5′-TGGTCCAGGGTTTCTTACTCC-3′
Mouse α-SMA (NM_007392)	5′-TGGCTATTCAGGCTGTGCTGTC-3′	5′-CAATCTCACGCTCGGCAGTAGT-3′
Mouse collagen I (NM_007742)	5′-GAGCGGAGAGTACTGGATCG-3′	5′-GCTTCTTTTCCTTGGGGTTC-3′
Rat GAPDH (NM_017008)	5′-TGTCACCAACTGGGACGATA-3′	5′-GGGGTGTTGAAGGTCTCAAA-3′
Rat α-SMA (NM_031004)	5′-GCGTGGCTATTCCTTCGTGACTAC-3′	5′-CATCAGGCAGTTCGTAGCTCTTCTC-3′
Rat collagen I (NM_053304)	5′-GCGTGGCTATTCCTTCGTGACTAC-3′	5′-CATCAGGCAGTTCGTAGCTCTTCTC-3′
Human GAPDH (NM_001256799)	5′-CAGGAGGCATTGCTGATGAT-3′	5′-GAAGGCTGGGGCTCATTT-3′
Human α-SMA (NM_001141945)	5′-TCCGGAGCGAAATACTCTG-3′	5′-CCCGGCTTCATCGTATTCCT-3′
Human collagen I (NM_000088)	5′-CCACCAATCACCTGCGTACA-3′	5′-CACGTCATCGCACAACACCT-3′
Mouse HO-1 (NM_010442)	5′-ACCGCCTTCCTGCTCAACATTG-3′	5′-CTCTGACGAAGTGACGCCATCTG-3′
Mouse NQO1 (NM_008706)	5′-GCGAGAAGAGCCCTGATTGTACTG-3′	5′-AGCCTCTACAGCAGCCTCCTTC-3′
Mouse p62 (NM_001290769)	5′-AGGAGGAGACGATGACTGGACAC-3′	5′-TTGGTCTGTAGGAGCCTGGTGAG-3′

## Data Availability

Not applicable.
